# Mental Health Nursing Students’ Perceptions of Psychological Trauma Education and Its Impact on Their Practice: A Qualitative Study

**DOI:** 10.3390/nursrep16020061

**Published:** 2026-02-13

**Authors:** Gwenne McIntosh, Margaret M. Conlon, Edel McGlanaghy, Freya Collier-Sewell

**Affiliations:** 1 Health Sciences Division, Faculty of Health Sciences and Sport, University of Stirling, Stirling FK9 4LA, UK; 2 NHS Forth Valley, Mayfield Building, Falkirk Community Hospital, Scotland FK1 5QE, UK; 3 Centre for Culture, Media & Society, Sheffield Hallam University, Sheffield S1 1WB, UK

**Keywords:** mental health nurse education, psychological trauma, wellbeing, trauma-informed education, trauma-informed practice

## Abstract

**Background:** Trauma-informed education (TIE) has become commonplace in nursing education; however, little is known about students’ experience of this and its impact on their practice. **Aim:** This study aimed to evaluate and explore mental health nursing students’ perspectives on TIE, and its impact on their practice, to contribute to the knowledge and evidence base that informs nursing and broader healthcare education. **Methods:** This qualitative, phenomenological study used a conversation café approach to focus group interviews (*n* = 3) with final year mental health nursing students (*n* = 11), reported using the Consolidated Criteria for Reporting Qualitative Research (COREQ) checklist. **Analysis:** The data generated was analysed using reflective thematic analysis. **Findings:** Three themes were identified: 1. a compass for practice; 2. mental health nursing: between paradigms; and 3. supporting personal development and wellbeing. Integrating TIE within nursing education can support students to adopt the principles of trauma-informed care (TIC) both personally and in their practice. Improved self-awareness, recognition of trauma and adopting self-care strategies were valuable in supporting personal resilience and wellbeing and in managing the challenges of mental health practice. **Conclusions/Recommendations:** TIE has the potential to have a positive impact on wellbeing; therefore, its integration should be considered for all healthcare programmes. Further interprofessional research is needed to establish the longer-term impact of TIE as students progress into their nursing careers.

## 1. Introduction

Psychological trauma refers to experiences where an individual is overwhelmed by harmful events and/or perceived threats [1]. Individual responses to traumatic experiences are impacted by factors such as the type of traumatic event, whether acute or chronic; individual factors such as age and stage of development; and supports available to the person afterwards [2,3]. The impact of psychological trauma has gained significant attention, with growing evidence that traumatic experiences can have long-lasting effects on people’s emotional, physical and social health [4].

There is a high correlation between a history of trauma and the development of Complex Post-Traumatic Stress Disorder (PTSD [5]. The World Health Organization (2022) [6] International Classification of Diseases (ICD-11) identifies PTSD and Complex Post-Traumatic Stress Disorder (CPTSD) separately and does not confine PTSD to a single event. Instead, the two are distinct but related trauma conditions requiring tailored interventions [7]. These developments in diagnosis have led to an increased need for trauma-informed approaches and interventions that acknowledge the impact of trauma on a person’s life and relationships, including recognition that feelings of shame, confusion and disconnection with past experiences prevent individuals from sharing, processing and recovering [8]. Mental health nurses are more likely to provide care to those who have experienced psychological trauma [9] and, therefore, are a professional group who could be ideally placed to integrate a trauma-informed approach into their practice. By providing safe and containing (trauma-informed) working relationships, mental health nurses can validate mental distress, reduce the risk of re-traumatisation and potentially support recovery [10].

Those drawn to mental health practice often have their own personal trauma histories. Indeed, in their review of relevant literature, Henderson et al. [11] concluded that mental health professionals were significantly more likely to have had personal experience of trauma which, in turn, increases their risk of secondary trauma.

Nursing education programmes provide an opportunity to embed foundational knowledge and skills to support the development of trauma-informed approaches. As TIE has been established in the BSc Nursing under study since 2020, an opportunity to evaluate its impact and, more specifically, its influence on clinical practice was available. This study provides valuable insights into the experiences of mental health nursing students as they approach the end of their education programme.

## 2. Background

Since 2015, Scotland has taken a proactive approach to the development of a national trauma training strategy, set out to integrate the knowledge and skills healthcare staff require to effectively support people who have experience of psychological trauma. This culminated in the launch of the National Health Service (NHS) Education for Scotland (NES) Knowledge and Skills Framework “Transforming Psychological Trauma” in 2017, and associated resources [12], now part of a suite of resources entitled the National Trauma Transformation Programme (NTTP).

Trauma-informed care (TIC) recognises the effects of trauma—the resilience of people in ‘surviving the surviving’ and the systemic factors [13] that can have a knock-on effect on life chances. The approach acknowledges the responsibility of healthcare services to provide an environment that creates physical and emotional safety, and which seeks to reduce re-traumatisation [3]. TIC links to individual, operational and strategic aspects of the care environment, processes, communication, therapeutic relationships, leadership and staff wellbeing [10].

Mental health nursing education in Scotland set out to embed the knowledge and skills outlined in the NTTP framework [12] to ensure nursing students meet “skilled” level practice by the end of their undergraduate programme. Prior to the framework, the mental health education team here at the [author’s university] had begun to develop its trauma-informed curriculum. The foundation was set around the six principles of TIC [3] held within the collaboratively developed 2016 nursing curriculum [14]. This started a paradigm shift that aimed to create a future workforce that would “promote radical change in culture and practice” [14] (p. 19). Yet, despite TIE being introduced into nursing education, little is known about its impact and value.

Since Young et al. [14] in 2019, the nursing education programme at the authors’ university has had TIE woven throughout each year, with a significant emphasis in year two, offering a unique opportunity to explore how this focussed education impacts clinical practice. For mental health nursing students, there is an increased focus within three field-specific modules in the programme, mapped to “skilled” trauma practitioner level by the end of the third year. The main aim of this curriculum thread, in line with the previous vision, was to promote change and improve the experiences of people who use mental health services.

Study Aim:

To evaluate the impact of TIE in an undergraduate mental health nursing programme and explore how mental health nursing students integrate knowledge and skills related to psychological trauma into their practice.

Research Questions:

What are mental health nursing students’ perceptions of their knowledge and skills related to psychological trauma?

How do mental health nursing students use their psychological trauma-informed knowledge and skills in their clinical practice?

How do mental health nursing students use their trauma-informed knowledge and skills to support their own wellbeing?

What do mental health nursing students perceive to be the barriers and facilitators to adopting a trauma-informed approach in mental healthcare environments?

## 3. Methods

This study adopted a qualitative phenomenological methodology to explore mental health nursing students’ experiences and perceptions of TIE within their undergraduate programme. Phenomenology focusses on meaning from the individual’s perspective, illuminating the essence of the experience from their point of view [15,16] and the meaning they construct [17]. Hermeneutic phenomenology has become established in health-related research, potentially due to its focus on the lived experience and individuals’ unique understanding of the world [18], being closely aligned with person-centred approaches to healthcare practice today [19,20].

This study included 3 online focus groups, held using MS Teams, which adopted a recovery conversation café (hereafter, ‘conversation café’) approach [21]. A total of 11 students participated in the conversation cafés. The recovery-informed approach of the conversation cafés aims to encapsulate principles of equal participation—potentially an issue with focus groups [22]—while promoting curiosity and dialogue, ensuring that everyone’s contributions are welcomed. A semi-structured focus group guide was developed to create a strengths-based and empowering environment. The methods adopted aligned with phenomenology and provided participants with the opportunity to share and explore, in a peer-to-peer setting, their experiences of TIE, leading to rich and deep discussions [23]. The rich data generated therefore required an analytic approach that would capture the complexity of human experience within a social context [24].

Research Team Characteristics

The authors comprised the research team and experienced qualitative researchers. Authors 1 and 2 were lecturers in the university and known to the participants; therefore, they were not involved in the conversation café facilitation to avoid bias or coercion. Authors 3 and 4 were conversation café facilitators and were not employed by the university. The primary analysis was conducted by Author 1, who is experienced in thematic analysis. In line with Braun and Clarke’s reflective thematic analysis guidance [24], the analysis was reflexive, with initial coding and themes shared and discussed in depth with the research team.

Context and Sample

This study was carried out in a higher education institution in the United Kingdom that delivers a three-year undergraduate mental health nursing pre-registration programme. A purposeful sampling approach was adopted. Invitations to participate were sent to all final year students on the BSc Mental Health Nursing programme. The inclusion and exclusion criteria are detailed in Table 1. All participants had completed the psychological trauma focussed education within the programme and undertaken at least one clinical placement following this education.

### 3.1. Recruitment

A total of 125 nursing students, across both the 2020 and 2021 cohorts BSc Nursing (Mental Health), were eligible to participate when in the final year of their programme. Following institutional ethical approval (Project ID 10629), study information was disseminated via announcements in the programme’s virtual learning environment and in class following module presentations and lectures. Contact information and links to study information and the consent form were provided to those interested. Once consent was confirmed, participants were asked to join an online conversation café.

### 3.2. Participants

The conversation cafés were held between May 2023 and May 2024. Eleven mental health nursing students participated, and one peer participant joined group one and two (see Table 2). There were two male and nine female participants, broadly in keeping with the demographics of the wider cohort.

### 3.3. Ethical Considerations

Ethical considerations were based on the Declaration of Helsinki [25]. Consent was obtained from all participants prior to each conversation café via an online form and, additionally, was verbally confirmed at the start of each conversation café. Participant confidentiality was maintained by ensuring participants details were stored electronically on the lead researcher’s password-protected laptop. Transcripts were anonymised and pseudonyms allocated.

The risk of personal disclosure—hearing about psychological trauma while in practice—was present. Facilitators received training on ‘hearing a disclosure’ from a member of the research team who is an expert in trauma-informed practice. Details of resources and support networks were shared with all participants after the conversation café, and the facilitator and peer participant engaged in reflexive discussions with the research team after the interview.

### 3.4. Data Analysis

Data from the audio recordings of the three conversation cafés were transcribed verbatim and analysed using Braun & Clarke’s [24] reflective thematic analysis. One researcher, experienced in thematic analysis, identified the initial themes and sub-themes through close analysis of the transcriptions, maintaining the focus on the lived experience, individual perspectives and meaning-making, aligning to the methodological approach. Through reflexive discussions with the wider research team, using the transcripts and coding, the final themes titles and sub-themes were consolidated and agreed upon.

## 4. Findings

Three themes were identified: 1. a compass for practice; 2. mental health nursing: between paradigms; 3. supporting personal development and wellbeing (see Table 3).

### 4.1. Theme 1: A Compass for Practice

The analysis of the data identified that TIE provided both a sense of direction—a compass—as well as an anchor in the form of a set of principles through which nursing students could understand, define and practice relationship-based, individualised care. Participants placed importance on developing skills to navigate clinical practice and viewed TIE principles, interpersonal skills and relationship-based approaches as the essential tools. A compass for practice engenders a sense of direction, an approach that helps nursing students move forward and identify aspects of mental health practice that require some navigation.

*“it’s harder for students when you go on a placement at your first day and you were like, ohh yeah, go do a one to one with this person, when actually you don’t know anything about them.”*

RC2 Nicole

However, tensions are evident in the comments from Emily, indicating that people may be disingenuous, and they may be viewed as being “taken advantage” of by others. Potentially showing some underlying stereotypical views of mental health service users:

*“… but it can be quite difficult sort of navigating sometimes … is this person being genuine right now, or are they sort of taken an advantage [of me] to degree, because of the type of person you are, does that makes sense?”*

RC1 Emily

There are also some risks in taking over simplified and generalised aspects of TIE, leading to students seeing everything through a ‘trauma lens’, to the exclusion of other perspectives:

*“… it really opened my eyes to the extent of the consequences of, you know, ACEs adverse childhood experiences and the impact on mental health but also physical health. Emm, And the the actual changes in the brain and emm hormones and things like that. Actually people’s brains act differently when they’ve been traumatised.”*

RC3 Grant

Sub-themes included participants drive to find strategies to develop their trauma-informed skills and knowledge and to promote a relationship-based way of working with others. Lucy described a holistic approach to trauma-informed skills to avoid harm:

*“I basically started asking people like, what happened to them like instead of asking what’s wrong or, you know, like, just to look at the person as a whole, not just as a as a diagnosis.”*

RC3 Lucy

Knowledge gained through TIE supported mental health nursing students to find approaches that promote positive relationships and that help avoid “causing harm”. The discussions in the conversation cafés highlighted that trauma-informed principles were being embedded in practice, whether a trauma history was known to be present or not. For example, the skills were being used to reduce the risk of re-traumatisation, to increase the importance of validation and create a sense of safety:

*“So it [TIE] gives us something to fall back on and say I know these principles work for everybody, no matter whether they’ve disclosed or not. So if I stick to these, there’s less chance of me causing this person harm.”*

RC2 Nicole

*“I think I was it in [placement] when we were on the trauma-informed module, and that definitely helped me go into the placement and use that trauma like … lens as they say. So you’re ‘okay what’s happened to you that’s lead you down that path’ as opposed to going ‘why are you using [substances]’?”*

RC2 Sophia

*“… because you don’t want to retraumatise people or just, you know, trying to be respectful and just, like, keep them safe.”*

RC3 Lucy

*“Do you know that people just even just asking you, you know, is this OK if I do this? Does this upset you or am I OK to come into your room?”*

RC3 Kim

This offered re-assurance for participants when connecting with people receiving mental healthcare and establishing relationships with members of the healthcare team:

*“I think it is also about the staff, some of the staff have their own experience as well so its recognising when you might have to step in or check in with.”*

RC2 Sophia

Importance was placed on the use of language, avoiding pre-judgement and recognising when others hold different views. The participants describe tensions between adopting a trauma-informed approach in practice:

*“It definitely did influence me because when you go out in practise, you hear people talking about trauma and how like some patients will have significant trauma in their background. But you it’s there’s, it’s never a weight on these words. So it’s just being mentioned. But like people just like, oh, she’s difficult. Yeah, yeah. She had loads of trauma in her life.”*

RC3 Lucy

Adopting a trauma-informed approach helped participants to identify barriers in practice and find ways to challenge these and promote positive change.

### 4.2. Theme 2: Mental Health Nursing: Between Paradigms

The data showed that healthcare systems, leadership, policies and procedures, and staff culture all influenced whether the participants perceived a workplace to be trauma-informed. The participants described tension between their education experience and the ‘real world’ (clinical practice). For some, this was explicit, with staff describing students as naïve, by people who are considered as being “at it”:

*“I do feel sometimes a bit disheartened when I I tried to implement that [TIE] into practise. It’s almost met with a bit of scepticism and, you’re a bit naive, you know. ‘They’re just at it’. That’s a common one. And or ‘it’s just behavioural’.”*

RC3 Lucy

*“you can sometimes be told ‘don’t engage with that person, don’t encourage you know they want your you want, they want you to, they want to suck you in and tell you everything and that’s not helpful for them’.”*

RC3 Rachel

However, TIE supported nursing students’ confidence to do things differently, even when negative views of adopting trauma-informed principles were held by senior staff:

*“And just things being softer and I think a lot of it would have to to be kind of maybe from the the top down as well. Do you know it’s just the culture of how you’re treated?”*

RC3 Kim

Some environments were felt to be more challenging than others when adopting trauma-informed principles, such as inpatient acute care:

*“… a trauma informed environment would be anything that was a flip side of a [an acute] ward because it’s like, so”, “just do the opposite, you know?”*

RC3 Jade

*“mean I think there are good intension, but I think it rarely happens on the acute ward that you get that quality time to actually ask the patient what is happening for you today.”*

RC3 Anna

Supervision, and a team culture of wellbeing, were recognised as protective factors; however, these were more likely to be visible and present in community settings:

*“I’m going on about the community so much, but it’s just because this is what I saw this [trauma informed practice] being done.”*

RC3 Jade

The participants recognised that where they encountered staff who were dismissive or unsupportive regarding adopting a trauma-informed approach, it impacted their motivation:

*“you can tell … I don’t know how to explain it but, there’s some people that you can have those conversations with like staff wise and others that you think there’s no point.”*

RC3 Rachel

These negative attitudes presented barriers to being trauma-informed. Such experiences resulted in nursing students feeling disheartened and unsure and generated self-doubt:

*“I think I’ve given up and avoid challenging the the culture that can be present in a lot of the wards which is sad in a way.”*

RC3 Grant

*“[some staff say] “You’re just feeding into their sort of attention seeking and manipulation” and I think that makes me feel quite unsure as to the best way to to approach like the way that we were advised at uni emm, is not really what happens in in practise” “it does leave me feeling a bit unsure, sometimes just. What are the staff going to say if I engage too much with this person?”*

RC3 Rachel

Supervision, both formal and informal, was indicated as maintaining a positive environment. Being able to talk and reflect on experiences in practice supported learning and self-awareness, creating a culture that is inclusive:

*“Supervision was something that can can be used to try and kind of help people, kind of like deal with that and still be able to kind of like just just so that they’re not getting burned out” “Or if something difficult had happened during like one of the groups or something like that, the team were all they were all amazing at it basically. So somebody would come in and say, listen, this happened and then everybody stop and … like a reflective group almost.”*

RC3 Jade

Participants recognised that environments where there were higher staff ratios supported trauma-informed approaches; however, there was acknowledgement that decisions about this were not usually within the control of nurses:

*“I think some of the things that are done in there [community] would be great if they could be done in an acute ward, but I appreciate there’s probably reasons why they aren’t happening. So there’s a maximum of 12 patients, but there’s a still the same number of staffing” “I’m not sure why, you know who decides that that ratio? I’m guessing that someone high up.”*

RC3 Rachel

### 4.3. Theme 3: Supporting Personal Development and Wellbeing

TIE, with its focus on mental wellbeing and self-awareness, encouraged participants to reflect on and value their own mental health. Nursing students described engaging in coping strategies—self-care activities to support their wellbeing, grow their self-awareness, personal and professional development and confidence levels:

*“I think that having this framework has probably saved a lot of people’s … a lot of students mental health as well. Because actually it has prompted us to think about ourselves.”*

RC2 Nicole

*“It’s crucial for any sort of trauma approach like it’s crucial for any sort of self-development or sort of self-understanding. So because sometimes like due to the trauma you can be really really toxic toward yourselves. And I think like the the very big step first step like is to learn what self compassion is. So definitely the the teachings from the the modules in that has has you know, I have been learning from that.”*

RC3 Grant

Personal wellbeing and resilience were viewed as important, with participants describing strategies that supported the emotional demands experienced. There was a combination of active processes where participants described purposefully reflecting on thoughts, emotions and reactions to experiences while running or walking:

*“… it has been quite good [walking] and not taking my phone with me or not listening to audio books, which is usually my kind of go-to, but just having the silence. So it’s been quite nice. And all your kind of thoughts come in then.”*

RC3 Kim

While some used physical activity, others used activities to distract and focus attention on other things as a strategy to stop thinking about their experiences. Although this could be considered as a form of avoiding the processing of difficult experiences, participants described these activities as helpful:

*“So that’s something [physical activity] that I do regularly. It’s quite intense, but I think it stops me thinking about things because you need to think about what you’re doing and it gets to the point where you’re just so tired that you can’t think about anything anyway, so that’s quite good. I quite like that.”*

RC3 Jade

Focussing on achievements and what has gone well encouraged positive thinking and recognition of personal strengths, creating an emotional ballast to draw on when times get difficult, ‘giving yourself that reassurance’, being able to buoy yourself up and keep afloat:

*“… taking that time to reflect and being like, right, OK, was there anything I done really, really well today, I know for a fact that that person probably felt a lot calmer after speaking to me.”*

RC1 Emily

There was recognition that working in the field of mental health practice is challenging, and acute mental health inpatient care, specifically, was described as frightening and chaotic:

*“But, it [acute care] was like hellfire in the ward and you were just consistently firefighting.”*

RC 1 Anna

*“You know, it’s frightening [acute wards]. It’s really frightening. I would be terrified.”*

RC3 Kim

Participants account of protecting their own wellbeing were set within the context of being able to engage with others in a trauma-informed way:

*“Emm, And … but you can’t pour from an empty cup, and it’s something that’s been kind-off drilled into us and I’m really glad it has because, as much as, the profession is challenging” “it’s very much about taking care of yourself first.”*

RC2 Sophia

Adopting trauma-informed principles both in their practice and their own life, encouraged participants to engage in reflection, increased self-awareness and helped them to recognise and connect with feeling overwhelmed so that they could take action to address this:

*“it’s actually one of the questions you ask yourself before you go to try and administer mental health first aid, is actually, ‘am I the best person that we doing this? Am I in the right space?’”*

RC2 Nicole

*“… it [TIE] definitely makes you reflect on your own experiences and you know the trauma that you maybe have experienced, things that you might not even … things could be particularly triggering for you [ ] I definitely have more self-awareness and I’m much more aware of, you know what things I I find difficult and the you know for me […] that definitely is contributed to me feeling a lot more resilient.”*

RC3 Rachel

The BSc Nursing programme was seen as challenging. Participants reported feeling overwhelmed by the many demands, limiting the time to look after their own wellbeing:

*“Like, I feel a bit overwhelmed lately with like, either having to work all the time and then when I’m not working, I’m basically studying so it is getting a bit too much.”*

RC3 Lucy

TIE supported confidence by providing a set of skills that can be used in different situations, with different people:

*“like I say, when you’ve got these tools, your confidence builds” “I think you’re confidence in what you’re doing.”*

RC2 Daniel

*“… [TIE] helps to inspire trust and hope between you and whoever you’re interacting with.”*

RC2 Nicole

## 5. Discussion

The findings indicate that the participants not only have good knowledge of the principles of trauma-informed practice, but also that they apply the skills in practice and in looking after their own wellbeing. However, applying trauma-informed principles depends on the environment, leadership, culture and attitudes held by staff.

TIE was indicated as providing a compass for navigating mental health practice. Participants connected TIE to supporting their confidence and skills to maintain a values-based and relationship-centred approach to their practice, in line with findings from Robinson et al. [9]. TIE is embedded throughout the nursing programme, in line with recommendations by Bosse et al. [26]; however, how this is sustained and maintained beyond pre-registration education is worth considering.

The participants in this study indicated that community settings demonstrated more engagement with trauma-informed principles and reflection. This was in stark contrast to acute inpatient settings, which were described as chaotic and not conducive to being trauma-informed. Wilson et al. [27] acknowledged that there was a lack of implementation of TIC in acute settings, citing the lack of adequate TIE, and the preparation of staff and the environment, as key barriers. This is evident in the data from this study, with participants reporting that their experience of acute care was at odds with what a trauma-informed environment needs to be. Heffernan et al. [28] report that TIE needs to be delivered in a cyclical and sustainable way and be open to all. This approach may bring opportunities to challenge stigmatising views and negative attitudes experienced. If TIC becomes embedded into pre-registration nursing education, this could result in the culture shift needed to improve experiences for all.

Heffernan et al. [28] propose that staff need to identify and make use of support networks. However, in this study, there is a sense that staff in acute care settings were “fire-fighting”; therefore, finding the time and space to engage in education and support may be challenging. Commitment from senior leadership is needed for TIP to be integrated into the current biomedical focused MH care system [28,29] if change is to be implemented and sustained. The participants in this study recognised the need for a system-wide approach to embedding TIC in acute inpatient care settings, and that strong leadership was key to this change.

Participants employ trauma-informed principles to reflect on, make sense of and manage their responses to the mental healthcare settings they are working in. Interestingly, the participants did not talk about needing to use trauma-informed skills to respond to the ‘felt trauma’ of the people using services, but that they do employ it to make sense of what is happening in terms of the practice environment and approaches they see nurses adopt. This contrasts with the literature demonstrating the impact of vicarious trauma on staff [9,30,31,32]. This focus on self and navigating practice may be linked to the participants’ student status, their focus on being assessed and the time-limited exposure to healthcare environments during practice placements.

For those working therapeutically with people who have experienced complex trauma, there is increased risk of vicarious traumatisation when compared to other care contexts [32]. Coleman et al. [32] and Bulford et al. [30] share the view that, given the increased prevalence of psychological trauma, clinical staff must be appropriately prepared and supported if they are to manage the emotional load of these interactions. Staff training is valuable [8]; however, it needs to be ongoing to sustain the benefits. Our study offers some insights into mental health nursing students’ strategies for self-care and how TIE may encourage more focus on self-care as a means of building resilience within the future mental health workforce. Mental health nursing students who have their own trauma experiences are at increased risk of re-traumatisation in mental health practice. This study has indicated that focussed education helped students to use TIC principles to protect their own mental health.

TIE appears to have encouraged participants to reflect on the healthcare system and their role within it. The analysis highlighted subtle tensions, such as the moves participants frequently make back and forth between diagnostic categories and biomedical understanding of mental health, and the psychological trauma-informed approach. This tension, also highlighted by Heffernan et al. [28], may reflect a tension within the healthcare system more generally. Working side by side with those who hold different perspectives can sometimes be complimentary, but if not managed well, can, by contrast, sometimes lead to unhelpful friction [33]. This shift in paradigm is discussed by Bulford et al. [30] in their literature review of primary care practitioners’ experience of TIC. They note that many primary care practitioners are experiencing a changing paradigm, where there is a move away from biomedical to more holistic explanations of health.

More broadly, there is a balance to be found so that TIE does not result in the next generation of mental health nurses proposing that people should, or must, make sense of their experiences through this lens. The implications (and unintended consequences) could be that we replace one fixed way of understanding ‘problems’ or ‘medical diagnoses’ with another, potentially imposing meaning onto others’ experiences in unhelpful ways [34] and limiting what is available to people in terms of how we imagine and make sense of our experiences. Nursing education, therefore, needs to reflect the range and complexity of how mental health and ill-health are understood.

Strategies supporting resilience included actively processing thoughts, emotions and ideas: walking, running, being outside and reflecting. Others adopted distraction: stopping thoughts, shifting focus, attention on something else, “switching off” and disconnecting with the emotional impact of mental health practice. The latter is potentially risky—for example, limiting the processing of emotions; however, these were recognised as valuable in helping participants find ways to create distance or separation between practice, study and life. Xiao et al. [35] discusses positive coping for nursing students. Confronting and resolving negative emotions is more likely to bring about increased resilience than avoidance, which may lead to greater susceptibility to its influence on wellbeing.

## 6. Conclusions

Overall, this small-scale study found that mental health nursing students feel confident in their knowledge and skills related to psychological trauma and reported applying the principles in their practice, following comprehensive and embedded TIE. The participants also indicated that they use trauma-informed knowledge and skills to support their own wellbeing, recognising that working in mental health settings and with distress can have an impact on their wellbeing and that their wellbeing can impact their clinical practice. Thus, employing trauma-informed selfcare was identified as a holistic way in which they can look after themselves and enhance the mental healthcare they provide to patients. There was recognition that different environments and team cultures in clinical practice affected participants’ wellbeing. TIE was cited as positive in relation to enhancing self-awareness, improving resilience and navigating challenging clinical practice. Further research is needed to establish the potential benefit of TIE in other healthcare programmes. Equally, there would be value in a longer-term study to provide evidence on the impact of TIE as students’ progress into their professional careers.

## 7. Recommendations

As TIE enabled mental health nursing students to identify and apply principles of trauma-informed care both personally and in their practice, it is recommended that TIE be embedded in healthcare education programmes more generally.

Inpatient acute care settings are challenging environments for trauma-informed principles and practice to be embedded in; therefore, a tailored approach to the education of staff in acute care settings would be of value.

Further research is recommended to better understand the impact of pedagogical and clinical practice components of nursing education programmes to better prepare students for mental health practice.

## 8. Limitations

Of 125 eligible mental health nursing students, 11 took part in conversation café sessions, and selection bias is important to consider. The high levels of agreement across participants indicate that social desirability may have influenced some participants.

Arranging the conversation cafés for students when on placement proved challenging (RC 1 and 2), resulting in poor recruitment. The final café was arranged when nursing students were in university, resulting in improved recruitment.

The research team was not able to seek feedback on the thematic analysis as once available; the students has completed their programme.

The use of the conversation café principles for the focus group is an innovative approach aimed at creating a relational and power neutral environment; this approach is new and may have influenced the narrative of each group discussion and, therefore, the data gathered.

This is a small-scale study within one university and is not intended to be generalisable.

## Figures and Tables

**Table 1:** Inclusion/exclusion criteria.

Inclusion Criteria	Rationale
September 2020 and September 2021 cohort, BSc/BSc Hons Nursing (Mental Health) students.	Completed the TIE components in the curriculum.
Completion of all mental health specific modules in programme	
Participants will have commenced at least one clinical placement in their final year of study.	Opportunity to apply, observe or experience trauma-informed theory and principles.
Exclusion Criteria	Rational
Adult health nursing students on the BSc/BSc Hons Nursing programme.	Do not have the same level of TIE in their programme.
All nursing students in first/second of the programme.	Have not completed the core trauma 3-year content.
Nursing students who have not progressed into clinical practice.	No opportunity to apply trauma knowledge in a mental health setting.

**Table 2:** Recovery conversation café details.

Recovery Conversation	Duration	Group Composition	Gender	Cohort
RC1	98 min	2 student participants 1 peer participant 1 facilitator	3 Female	September 2020
RC2	68 min	4 student participants 1 peer participant 1 facilitator	4 Female 1 Male	September 2020
RC3	90 min	5 student participants 1 facilitator	4 Female 1 Male	September 2021

**Table 3:** Themes and sub-themes.

Theme	Sub-Themes
Theme 1: TIE: A compass for practice	Offers a toolkit of knowledge and skills for working with others Helps identify and navigate barriers in practice Emphasises relationship-based approaches to move forward Provides direction for values-based, individualised and relationships-based care Gives a framework to drive change
Theme 2: Mental health nursing: Between paradigms	TIP finding its place in the healthcare system The impact of negative attitudes Leadership plays a part Creating a positive culture for TIP The unique challenges of acute mental healthcare
Theme 3: TIE: Supporting personal development and wellbeing	Recognising demands on own mental wellbeing A better understanding of themselves Increased self-awareness and personal and professional self Encouraged self-care and looking after self Builds self-confidence and resilience

## Data Availability

Data for this study is unavailable due to privacy and ethical restrictions.

## Public Involvement Statement

The data collection methods used in this study were informed by people with lived experience; former students informed the early stages of this study and the interview questions.

## Guidelines and Standards Statement

This manuscript was drafted against the COREQ Checklist (Consolidated Criteria for Reporting Qualitative Research) [36].

## Abbreviations

The following abbreviations are used in this manuscript:

TIE	Trauma-informed education
CPTSD	Complex Post-Traumatic Stress Disorder
PTSD	Post-Traumatic Stress Disorder
TIP	Trauma-informed practice
